# How does slum rehabilitation influence appliance ownership? A structural model of non-income drivers

**DOI:** 10.1016/j.enpol.2019.06.005

**Published:** 2019-09

**Authors:** Ramit Debnath, Ronita Bardhan, Minna Sunikka-Blank

**Affiliations:** a Behaviour and Building Performance Group, Department of Architecture, University of Cambridge, CB21PX, UK; b Centre for Research in Arts, Social Sciences and Humanities, University of Cambridge, CB39DT, UK; c Sustainable Design Group, Centre for Urban Science and Engineering, Indian Institute of Technology Bombay, 400076, India

**Keywords:** Appliance ownership, Residential electricity, Slum, India, Energy efficiency, Poverty, Slum rehabilitation, Practice-based approach, SEM

## Abstract

This study explores the effect of slum rehabilitation on appliance ownership and its implications on residential electricity demand. The low-income scenario makes it unique because the entire proposition is based on the importance of non-income drivers of appliance ownership that includes effects of changing the built environment (BE), household practices (HP) and appliances characteristics (AC). This study demonstrates quantitatively that non-income factors around energy practices influence appliance ownership, and therefore electricity consumption. The methodology consists of questionnaire design across the dimension of BE, HP and AC based on social practice theory, surveying of 1224 households and empirical analysis using covariance-based structural equation modelling. Results show that higher appliance ownership in the slum rehabilitation housing is due to change in household practice, built environment and affordability criteria of the appliances. Change in HP shifts necessary activities like cooking, washing and cleaning from outdoor to indoor spaces that positively and significantly influences higher appliance ownership. Poor BE conditions about indoor air quality, thermal comfort and hygiene; and product cost, discounts and ease of use of the appliances also triggers higher appliance ownership. The findings of this study can aid in designing better regulatory and energy efficiency policies for low-income settlements.

## Highlights

• Slum rehabilitation causes household practices to shift indoors.
• Shifting of household practices to indoors increase appliance ownership.
• Slum rehabilitation increases energy intensive activities.
• Non-income factors around energy practices influence appliance ownership.

## 1 Introduction

Residential electricity demand is going to increase in the Global South with its accelerated economic growth in the coming decades. The International Energy Agency (IEA) states that OECD countries account for 65 per cent of the total residential electricity consumption globally, while electricity demand in non-OECD countries, especially in the Global South, have grown at twice the rate compared to the OECD countries (IEA, 2009). It is expected that non-OECD countries will exceed OECD countries’ demand by up to 25 per cent in 2030 (Cabeza et al., 2014). Global climate and energy scenario estimate future appliance penetration by using income (average national GDP), electrification and urbanisation driven logistic curves (McNeil et al., 2013; Daioglou et al., 2012; Farsi et al., 2007; McNeil and Letschert, 2010). This method is rooted based on the assumption that all households globally at a certain income level would have the same appliances which oversimplify reality (Rao and Ummel, 2017). However, factors like market accessibility, affordability, household characteristics, wealth and income levels together better explain appliance ownership (Rao and Ummel, 2017).

Everyday activities of occupants shape their need for household appliances which influence energy consumption. These activities represent practices, which are routinised type of behaviour of normal life (Reckwitz, 2002). These practices, consciously or not, drive the ‘practical rational’ behind appliance purchase decisions of individuals and households (Røpke, 2009). Practice-related research demand examinations of broader social processes that place the onus on the practices and how they are performed by households, instead of individuals as energy consumers or appliance users (Foulds et al., 2016). Social practise theory (SPT) is a tool to study such social processes. It states that practices are entities made of material arrangements (i.e. materials, technologies and tangible and physical entities), know-how and routines, institutionalised rules and teleoaffective structures (domains of symbols, meanings, beliefs and emotions) (Labanca and Bertoldi, 2018). Shove et al. (2012) conceptualised this idea for energy studies through the combinations material, meaning and competencies that together make up the social practice and shape the practice in their process of interaction. In SPT individuals act as a carrier of a practice which ultimately leads to decision-making rather than just the behavioural attributes. Shove et al. (2012) elucidation on the material dimension (i.e. objects, infrastructure, tools, hardware and the human body) of SPT establishes connecting theories around energy consumption in households, but the causations are yet to be studied. A change of built environment in low-income communities is linked with a change in their social processes. However, the influences of this change on the broader physical systems like energy remains understudied in the literature.

Known drivers of residential energy use at a macro-level are income, climate, demographic characteristics, along with energy price dynamics, dwelling type and technology evolution including information and communication technologies (ICT) (Cabeza et al., 2014), (Rao and Ummel, 2017), (Ekholm et al., 2010). In India, macro-level studies have revealed a hierarchy in the order in which goods are acquired (Daioglou et al., 2012). Both rural and urban households have been shown to follow complex energy transition trajectories and tend to rely on more than one energy source, as contrary to the idea of climbing the hypothetical energy ladder (Van Der Kroon et al., 2013). With the rise in household income, improved solutions become more accessible; there is a tendency to stack multiple energy sources, termed as ‘energy stacking’ or ‘energy staircase’ (Van Der Kroon et al., 2013; Kowsari and Zerriffi, 2011; Bisaga and Parikh, 2018). The accessibility of an ‘improved solution’ is shaped by the social practices of the occupants which adds meaning and competence to their daily life through appliance purchase (Bisaga and Parikh, 2018). As the improved solution complies with the household practices of a social class, appliance ownership increases, as occupants find value in owning that appliance. Practice theory suggests that appliance ownership is more context-specific than merely a function of income, and not everyone may acquire appliances in the same way as income rises (Shove et al., 2012).

In this study, we investigate whether the change of the built environment of slum dwellers to slum rehabilitation housing (SRH) affect their residential electricity use. In doing so, we investigate the number of appliance purchase in the slum rehabilitation housing after shifting and assume it as an indicator of end-user demand for residential electricity. This system boundary defines the novelty of this work, where we link change in built environment, household practice and appliance characteristics as essential drivers of appliance ownership in low-income settlements.

The policy of ‘Slum Rehabilitation Housing’ was adopted in 1995 by the Government of Maharashtra as a response to redevelop the slums into high-rise social housing by incentivising the private sector to participate in the redevelopment of slum communities. The process mandated to re-house the slum dwellers onsite in high-rise buildings that includes legal entitlement to a stipulated 269-square-foot apartment, including a bathroom with tap water and a kitchenette. This policy provided the slum dweller access to a cross-subsidised, free of cost house, without burdening their time or economic poverty (Nutkiewicz et al., 2018; Debnath et al., 2017). The housing units though provided the slum dwellers with a permanent shelter. The units lack basic guidelines for design, energy efficiency or socio-cultural considerations (Sunikka-Blank et al., 2019). How housing units are used determines the dynamics of social practices that influence appliance ownership vis-à-vis energy use.

We base our study in an SRH society in Mumbai that comprises of occupants who have been living here for more than six months. Previous studies have shown that these SRHs often resemble ‘vertical slums’ characterised through the hyper-density of occupants, sub-standard dwelling quality and inadequacy to provide basic amenities (Bardhan et al., 2018a, 2018b; Debnath et al., 2019; Zhang, 2016). These sub-standard conditions compel the occupants to move back to horizontal slums. A recent study has shown that about 40% of that occupants move out (Nair, 2018). In a similar study, it was found that higher appliance ownership in the SRH causes economic distress that causes wider psychological and social discomfort (Debnath et al., 2019). However, none of these studies investigated the effect of household practice on occupants' distress and discomfort in the SRH. The present study contributes to this gap in energy research and present empirical evidence on the influence of household practice and built environment on appliance ownership. The contribution of this study is twofold. First, it empirically links household practice, built environment and appliance characteristics to appliance ownership in low-income settlements under urban transition (slums to slum rehabilitation housing). Second, the policy implications of this study can guide in designing better subsidy and energy efficiency plans for such low-income communities.

**Fig. 1:**
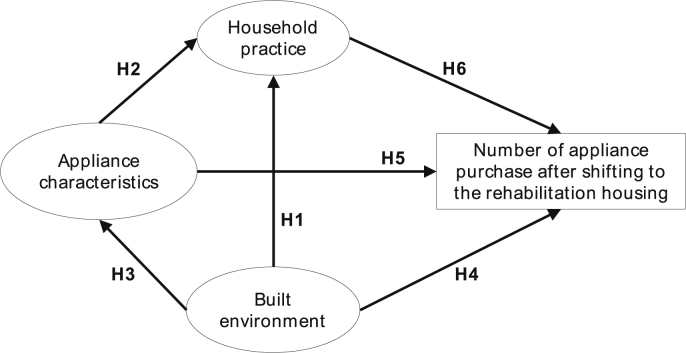
Conceptual model of the study indicating possible linkages between the variables.

The primary hypothesis of this study is that higher appliance ownership in the slum rehabilitation housing is due to change in household practice triggered by the change in the built environment from the horizontal slums to SRHs. Six sub-hypotheses (H1 to H6) were developed based on a conceptual model of appliance ownership in the rehabilitation houses. It is discussed in detail in Section 3.2 and Fig. 1. A questionnaire survey was developed to allocate the built environment, appliance characteristics and household practice-based questions to empirically test this hypothesis using a latent-variable modelling approach (see Section 3.1). The findings of the empirical analysis are illustrated in Section 4.2 preceded by a detailed presentation of the data descriptive (see Section 4.1). Policy implications of this study are offered in the discussion section (Section 5), and a broader policy pathway for energy sustainability in such urban transition is illustrated in the conclusion section (Section 6).

## 2 Background

Appliance ownership has mainly been discussed or predicted using empirical research based on societal trends, while very few studies examined determinants of appliance ownership at a household level (Rao and Ummel, 2017). Macroeconomic studies have estimated the household appliance penetration based on logistic curves driven by income, electrification and urbanisation (McNeil et al., 2013), (Daioglou et al., 2012), (Batih and Sorapipatana, 2016). However, these estimates do not explain historical appliance diffusion or investigate the household-specific factors that arise from coordinated and independent ‘homely’ practices that a household performs. Recent studies that describe the growth of household appliances in urban India and China are also skewed by only using the demographic characteristic data (especially the household size), and not considering the effect of household-level drivers in appliance ownership decisions (Cabeza et al., 2014), (Bhattacharyya, 2015). Studies that examined the effect of income elasticity on appliance purchasing capability discussed heavily between the patterns of income and energy growth (Wolfram et al., 2012; Auffhammer and Wolfram, 2014).

Among the few studies of household level determinants, most of the studies have reported household size to be a significant non-income determinant, along with housing type and age (O'Doherty et al., 2008; Leahy and Lyons Sean, 2010). In rural China, Rong and Yao (2003) found that more education, public services and a higher number of female members in a household increase the likelihood of appliance ownership. In rural India, Kemmler (2007) found that important non-income drivers were household characteristics, the degree of community electrification, and the quality of electricity supply. In urban India, Tiwari (2000) found that household size has positive significance on residential electricity consumption. It was recognised that a five-member family in Bombay (now Mumbai) would have 23% more electricity expenditure compared to a two-member family, an addition of an extra member increases the usage by 7.7%. Contrary to this study, Filippini and Pachauri (2004) determined negative correlation with electrical energy consumption in urban Indian households, stating that houses with more than six members had lower electricity consumption than those with fewer numbers. A study by Louw et al. (2008) in a low-income household in South Africa had established that the number of household members did not affect the electricity consumption as most of the electrical end-uses (like cooking or watching TV) were shared simultaneously between the occupants.

A significant effect of family composition (i.e. presence of children teenagers, adults and older adults) and the age of the household responsible person (HRP) has been widely acknowledged in the literature as a crucial non-income driver of energy consumption (Jones et al., 2015). However, these studies do not specifically comment on the drivers of appliance purchase, but rather comment on the effect of household energy consumption and the age of HRP. Tenure type of the house is also identified to have a significant impact on the total residential electricity consumption, but it remains a local-effect and differs widely with the study area (Jones et al., 2015; Wyatt, 2013). The relationship between dwelling type and higher appliance ownership remains unclear, as in general, the literature suggests that the influence of dwelling type on electricity consumption is related to the differences in floor area (Wyatt, 2013). Similarly, for dwelling age it is reported that newer homes have higher energy consumption despite owning higher energy efficient appliances; it is primarily attributed to the rebound effect of energy efficiency (Tiwari, 2000; Halvorsen and Larsen, 2001; Louw et al., 2008; Jones et al., 2015). However, most of these studies were conducted in developed countries and may be entirely different for a country like India where culture specific practices matter in driving the electricity demand (Sunikka-Blank et al., 2019).

Other commonly discussed built-environment elements that influence energy consumption are the number of rooms, bedrooms, floors and the floor area in addition to the presence of electric space heating, ventilation, electric hot water heating systems and air-conditioning systems (Jones et al., 2015; Brounen et al., 2012; Larsen and Nesbakken, 2004). As mentioned earlier, these studies report the findings from developed economies, in our study area, i.e. the slum rehabilitation housing, the presence of these elements (like mechanical cooling, ventilation, hot water system and space heating) is highly unlikely as the general population belong to the lower to middle-income demographic characteristics.

Rao and Ummel (2017) in their cross-country and micro-level study of Brazil, India and South Africa have shown that there is a significant variation in appliance penetration at a given income level, implying that country-specific factors matter. Upfront purchase cost that determines the affordability of an appliance matters most in low-income households across these countries. Appliances like television and refrigerators have higher penetration in all three countries, whereas the penetration of washing varies widely across the nations. Higher penetration of television and refrigerators can be explained by the influence of social practices with these appliances. While television (an Information and Communication Technology (ICT) equipment) provides a medium for entertainment and knowledge sharing, a refrigerator provides extra utility time, especially to the women of the house (Dhanaraj et al., 2018). These devices are purchased for use in households in order to contribute to customers' well-being by creating value in the form of knowledge or entertainment (in case of ICT devices), or by saving time from daily grocery shopping (in case of refrigerators) (Pothitou et al., 2017). Studies have reported that occupants tend to own the appliance which complements their social practices, which in turn influence their social practices (Pothitou et al., 2017; Røpke and Christensen, 2012). These appliances enable the users to save time from their day-to-day activities that enable them to derive greater utility from its usage. This extra time is very crucial for the low-income population as they often use it for income generation activities, especially in emerging economies (Sunikka-Blank et al., 2019). Therefore, ‘time-saved’ from ICT usage for economic activities is a primary cause behind its higher penetration rate in the emerging economies as compared to the OECD nations (IEA, 2009).

In another study by Dhanaraj et al. (2018), examining the reason behind low refrigerator ownership in India, the authors found that lack of female education and decision-making capability in the households significantly affect refrigerator purchasing behaviour in the household. The authors suggest that income is not a sufficient condition behind refrigerator ownership; the duration of electricity for more than 17 h a day has more significance. They also mention that as the females in the households tend to derive greater utility from refrigerator usage, their role in purchasing decision-making is vital. Refrigerator lowers household burden of work and eases women's entry into the labour market (Dhanaraj et al., 2018). Thus, appliances in such low-income settings have a dynamic role in changing practices and household welfare. Additionally, non-income drivers include household size, years of education of the HRP, number of rooms, dwelling quality, affordability, automobile ownership, female to male ratio, rural-urban migration, race and tenure type (Rao and Ummel, 2017).

While these drivers represent the material aspect of Social Practice Theory (SPT), the meaning and skills represent the abstract element of the occupants' socio-cultural dynamics that drives their energy stacking behaviour. In a study by Khalid and Sunikka-Blank (2017) in middle-class households in Pakistan have reported that material and social constructs of ‘homely’ household practice related to comfort, lighting, cleanliness, cooking and ICT were critical in driving ‘uncanny’ residential electricity demand. The modernistic prefiguration of spaces and electrical appliances shape occupants social practice, which in turn, shapes everyday energy practices (Khalid and Sunikka-Blank, 2017). In a similar work by Bisaga and Parikh (2018) in South Africa have reported that social practices change dramatically and depend on the availability of appliances. They found that appliance ownership does not increase linearly with time. Instead, the change in practices by using an appliance motivates occupants to purchase solar energy appliances. Foulds et al. (2016), investigated the trend in appliance purchasing amongst occupants who changed home to energy-efficient houses in the UK have found that moving home can change the appliance requirements. The social-meaning of specific appliance-using practices (e.g. stylishness convenience, thermal comfort, cleanliness) were significant in motivating occupants to purchase appliances. However, skills and competence to perform appliance-using practices were less prominent in influencing appliance ownership changes. They suggested that change in the built environment of occupants change appliance ownership based on appliance-using practices.

Rao and Ummel's (Rao and Ummel, 2017), Bisaga and Parikh's (Bisaga and Parikh, 2018) and Foulds et al.‘s (Foulds et al., 2016), studies provide critical clues for this work as the occupants living in the slum rehabilitation housing have heterogeneity in all the above drivers. These inhabitants are part of one of the biggest informal economy of the world (Debnath et al., 2017), they are rural to urban migrants, and they tend to restore their rural identity and social practices (Nijman, 2015). They live in low-income settlements, yet they have middle-income household characteristics (Zhang, 2016), these occupants own their houses which is a significant fulfilment of aspiration for the middle class (Bardhan et al., 2015). Moreover, these housing units are characterised by sub-standard living conditions in a vertical and high rise building fabric (Debnath et al., 2019). A study by Debnath et al. (2019), have reported that these occupants tend to move out of these houses, and rebound to their original horizontal slums. At present, it is estimated that nearly 40% of the inhabitants rebound by either selling or renting their SRH flats to a third person (Nair, 2018). The reason behind such a rebound behaviour was found out to be increased discomfort and distress in the slum rehabilitation households (SRH) owing to a mismatch between social, cultural and architectural design requirements of the occupants (Debnath et al., 2019). It was also due to higher appliance ownership among the rehabilitated occupants as they feel SRH as rising their social ladder, and they buy appliances as soon as they move in to fulfil their aspirations (Debnath et al., 2019). The transition of the built environment from slums to a permanent vertical housing structure (SRH) was found to influence their social practices that may have had a considerable impact on the appliance purchasing decisions, leading to higher electricity usage. However, Debnath et al. (2019) were inconclusive regarding the influence of changing the built environment on social practices that can lead to higher electricity consumption. In this study, we investigate these linkages and derive critical inferences to inform policies for sustainable low-income built environment and energy transitions in the Global South.

## 3 Data and method

### 3.1 Survey design

Mumbai has an estimated population of 12.4 million as per 2011 Census data, out of whom 42 per cent (i.e. 5.2 million) of the population lives in the slums (Zhang, 2016). Since the past two decades, 0.15 million slum dwellers have been rehabilitated into slum rehabilitation housing (SRH) by a state-owned body, Slum Rehabilitation Authority. Four slum rehabilitation housing (SRH) societies in the ‘Ward-M’ of Mumbai were selected for conducting the household surveys. It is estimated that more than 6,000 families live in these four SRHs.

The surveys spanned across 1,224 households which were selected using a stratified random sampling method across the four SRHs. A survey questionnaire was developed comprising of three segments that recorded the change in household practices on moving to SRH from the horizontal slums, perception of built environment and household appliance ownership criteria. The questions on the change in household practices were drawn after the work of Khalid and Sunikka-Blank (2017) which investigated the connections between ‘homely’ practices and its impact on high electricity demand in Pakistan. This study was explicitly referred to because of its similarity to the socio-cultural context of our study area.

A recent study by Sunikka-Blank et al. (2019), in the SRHs of Mumbai, had demonstrated the importance of women as critical household energy-decision makers. It guided our questionnaire formulation of household practices that can influence appliance ownership (see Table 1). A dichotomous variable *(1= Male, 0 = Female)* was included to understand the gender dynamics in the appliance-purchase decision. The interviewees were asked, ‘*Who usually makes the appliance purchase decisions?*‘. Other variables in this segment included documentation of the time-related mandatory household activities^1^ like cooking, washing and cleaning; as well as ICT usage activities that included time spent in watching TV and time spent in economic generation activities (i.e. work from home). These variables were reported on an ordinal scale *(1= Less than 1 h, 2= 1–2 h, 3= More than 2 h)*. Further narratives were collected based on the methodology of (Sunikka-Blank et al., 2019) to get a subjective overview of the household practices concerning energy usage in the horizontal slums and the SRH.

**Table 1:** Descriptive of the variables.

Survey variables	Parameter	Variable type	Mean	Std. dev	Min	Max
Number of appliances purchased after shifting	V1	Continuous	2.26	1.63	0	10
**Household Practice**						
Gender dynamics in appliance purchase decision making	P1	Dichotomous (Male = 1; Female = 0)	0.41	0.49	0	1
Hours spent in performing mandatory activity - Cooking	P2	Ordinal (Less than 1 h=1; 1 -2 h = 2; More than 2 h = 3; Not performing the activity = 4)	1.93	0.61	1	4
Hours spent in performing mandatory activity - Washing	P3	Ordinal (Less than 1 h=1; 1 -2 h = 2; More than 2 h = 3; Not performing the activity = 4)	1.56	0.67	1	4
Hours spent in performing mandatory activity - Cleaning	P4	Ordinal (Less than 1 h=1; 1 -2 h = 2; More than 2 h = 3; Not performing the activity = 4)	1.95	0.62	1	4
Hours spent in subsistence activity	P5	Ordinal (Less than 1 h=1; 1 -2 h = 2; More than 2 h = 3; Not performing the activity = 4)	3.71	0.73	1	4
Hours spent in ICT use including Television	P6	Ordinal (Less than 1 h=1; 1 -2 h = 2; More than 2 h = 3; Not performing the activity = 4)	2.58	0.85	1	4
Increase in number of activities performed indoors after shifting	P7	Continuous	0.49	0.71	0	4
Increase in energy intensive activities	P8	Dichotomous (Yes = 1; No = 0)	0.45	0.50	0	1
**Built environment**						
Perception of Indoor Air Quality	BE1	Likert scale (Very poor = 1; Poor = 2; Average = 3; Good = 4; Very good = 5)	3.26	0.90	1	5
Concern regarding hygiene	BE2	Dichotomous (Yes = 1; No = 0)	0.73	0.44	0	1
Perception of thermal comfort in rehabilitation house	BE3	Likert scale (Very cold = 1; Cold = 2; Slightly cold = 3; Neutral = 4; Slightly hot = 5; Hot = 6; Very hot = 7)	4.16	1.41	1	7
Perception of thermal comfort in comparison to the horizontal slums	BE4	Ordinal likert (Same= 0; Less comfortable = 1; More comfortable = 2)	1.28	0.69	0	2
**Appliance characteristics**						
Size	A1	Dichotomous (Yes = 1; No = 0)	0.73	0.44	0	1
Brand	A2	Dichotomous (Yes = 1; No = 0)	0.81	0.39	0	1
Product cost	A3	Dichotomous (Yes = 1; No = 0)	0.88	0.33	0	1
Quality	A4	Dichotomous (Yes = 1; No = 0)	0.86	0.35	0	1
Discount available	A5	Dichotomous (Yes = 1; No = 0)	0.84	0.37	0	1
Ease of use	A6	Dichotomous (Yes = 1; No = 0)	0.67	0.47	0	1

Additionally, based on appliance ownership in the current housing, a dichotomous variable was introduced to report the change in energy-intensive practices. It was assumed that if the household bought energy-intensive appliances like TV, washing machine, microwave, refrigerator, computer, laptop/tablet computers and clothing irons, then there was a significant shift in energy-intensive practices, and it was coded as ‘*Yes = 1’ or else ‘No = 0*’. A continuous variable on the increase in the number of activities performed indoors was also reported (See Table 1). Mandatory household activities like cooking, cleaning and washing were mostly performed by female members of the households during the day, as the male (working-age) members remain out-of-house for work (Sunikka-Blank et al., 2019). It characterises a critical social norm of these low-income settlements that the working-age male member spends the daytime out of the house (12–18 h/day). Therefore, household practice-related questions were directed to the female members of the household.

Questions relating to the built-environmental attributes were derived from the recent study of Bardhan et al. (2018b), (Bardhan et al., 2018a) that indicated indoor air quality (IAQ), hygiene and thermal comfort to be a crucial driver of occupant dissatisfaction in low-income tenement houses (see Table 1). The occupants were asked to rate their IAQ using a Likert-like scale consisting of five *parameters (Very poor = 1; Poor = 2; Average = 3; Good = 4; Very good = 5)*. Similar scale was used to report the occupant's perception of the thermal comfort in their present rehabilitation *houses (Very cold = 1; Cold = 2; Slightly cold = 3; Neutral = 4; Slightly hot = 5; Hot = 6; Very hot = 7)*. Additionally, an ordinal variable was added to report the change in thermal comfort conditions as compared to the horizontal slums from the occupant's perspective and was coded as ‘*Same= 0; Less comfortable = 1; More comfortable = 2*’.

Variables related to appliance characteristics were based on the consumer culture of appliance ownership in India, as shown in the work of Eckhardt and Mahi (2012). Six dichotomous variables *(1 = Yes, 0 = No)* defining product characteristics was used that included size, brand, product cost, quality, availability of discount and the ease of using the appliance (see Table 1). These product variables cumulatively indicate the affordability criteria; however, ‘affordability’ is not explicitly analysed as a separate indicator in this study.

To link drivers of appliance ownership with the built environment and household practices, an observed continuous variable was introduced in the survey questionnaire that recorded the number of appliances purchased after shifting into the SRH. This indicator was treated as the endogenous variable for the analytical model (see Fig. 1) and is discussed in detail in Section 3.2. The variables and descriptive are illustrated in Table 1.

### 3.2 Hypothetical model development

Six hypotheses were formulated based on the evidence from the literature on social practice and residential electricity use, built environment and its effect on energy demand and appliance ownership characteristics in low-income tenement housing (refer to Section 2). Additionally, increased appliance ownership after shifting to SRH was considered as an endogenous variable. These hypotheses are illustrated in the conceptual framework in Fig. 1. They are represented as H1, H2, H3, H4, H5 and H6, respectively.

H1 Shifting of occupants from horizontal slums to vertical slum rehabilitation housing has a positive effect on change in household practice.

This hypothesis is based on recent studies in Lahore, Pakistan (Khalid and Sunikka-Blank, 2017) that showed a change in built environment design of the occupants had a positive impact on high residential electricity consumption through a change of household practice. It is further supported by Rao and Ummel's (Rao and Ummel, 2017) derivation of non-income drivers of appliance ownership in India, Brazil and South Africa that listed dwelling type and the number of rooms as critical built environment variables affecting practices that in turn influence appliance ownership. Policy insights from social practice theories on energy conservation by Labanca and Bertoldi (2018) also support this hypothesis.

This hypothesis tests that when occupants move from horizontal slums to vertical slum rehabilitation houses, it has a positive effect on change in household practice. In horizontal slums, the social practices are mostly associated with the outdoor social spaces, like, cooking, washing clothes, cleaning and social interactions with neighbours (Sunikka-Blank et al., 2019). Such communal spaces are missing in the vertical slum rehabilitation housing that may influence the change of household practices.

H2 Appliance characteristics have a positive effect on the change in household practices.

H2 is supported by the findings of Bisaga and Parikh (2018) that showed that change of practice of rural households in Rwanda had a positive non-linear influence on solar home system adaptation due to increase in energy demand in the households. The author's mentioned that change of practice due to certain solar appliance ownership further motivated the occupants to invest in solar energy solutions. In another study by Dhanaraj et al. (2018), the effect of refrigerator ownership in low-to-middle income households in India was presented. The authors showed that introduction of refrigerator contributed to the welfare of female members of the households through a change of household practices.

H3 Transition of the built environment from horizontal slums to vertical slum rehabilitation housing has a positive effect on choosing specific appliance characteristics.

H3 is supported by a recent study by Foulds et al. (2016), that showed that shifting house influences appliance ownership. This study was conducted for recent Passivhaus (energy efficient homes) owners in the UK and showed that high energy efficiency status of the houses influenced higher appliance ownership, that in turn resulted in higher usage time and energy demand. Extended evidence can be found in a review paper by Janda (2011).

In case of the slum rehabilitated households, the shift from the slums entails the idea of climbing up the social ladder. The shifting of the occupants from a temporary slum to the permanent SRH may instil the aspiration to purchase more appliances, as permanent housing is associated with the notion of moving out of poverty. This aspirational component influences the occupants to choose specific characteristics that complement the perception of climbing up the social ladder like the brand, size, product cost and quality (Eckhardt and Mahi, 2012). Contrastingly, in horizontal slums occupants preferred to purchase unrated appliances to save in upfront cost (Khosla and Bharadwaj, 2015). Additionally, the space constraint in the slums restricted the uptake of appliances which was relieved in the SRH.

H4 Change in the built environment has a positive effect on the number of appliance purchase after shifting to SRH.

It is a further extension of H3, where the change of the built environment represents the transition of the slum dwellers from the informal shacks to permanent high-rise apartments in the slum rehabilitation houses. As mentioned earlier, aspiration and the feeling of climbing up the social ladder plays a crucial role in determining the purchasing decision of new appliances having specific lifestyle characteristics (like the brand, size, cost and quality). The hypothesis H4 examines the quantitative aspect of the effect of built environment transition on the appliance ownership in the SRH. The change of the built environment in the SRH is marked by the lack of outdoor social spaces that compels the occupants to spend more time indoors, as contrary to the living in horizontal slums. Literature shows that such transition also influences household practices that can, in turn, affect the household electricity demand (Foulds et al., 2016).

H4 has its basis on the findings of Foulds et al. (2016), where they established a positive influence of energy efficiency house on the appliance ownership. However, the setting of the study was in a higher income group, unlike this study. Similar inferences were also drawn by Rao and Ummel (2017) on dwelling type as a positive influencer of appliance purchase of occupants on a macro and regional level dataset of India, Brazil and South Africa. Khalid and Sunikka-Blank (2018) showed that the change of built environment in middle-income households in Lahore, Pakistan increases energy demand due to the change of practice. It is a close representation of our case study in the SRH of Mumbai, India and forms a critical lead for the conceptualisation of H4.

H5 Appliance characteristics have a positive effect on the number of appliance purchase after shifting to SRH.

Hypothesis 5 (H5) is built on the assumptions of classical macroeconomic and consumer theory that appliance characteristics like quality, brand, size, cost and discount available significantly influence appliance ownership across all socio-economic classes (Filippini and Pachauri, 2004).

H6 Change in the household practice of the occupants has a positive effect on the number of appliance purchase after shifting to SRH.

H6 is assumed based on the recent advancement in the sociological consumption studies based on practice-theory approach. Practices are meaningful to people, and people are influenced by the practices they are engaged in everyday life (Røpke, 2009). Consumption comes in as an aspect of practices (discussed in detail in Section 2). This hypothesis adds to the merit of the study, as for the first time we are saying that in low-income households, appliance ownership is influenced by household practices and changing built environment. Other literature-based evidence in support of H6 can be found in (Foulds et al., 2016), (Khalid and Sunikka-Blank, 2018), (Sunikka-Blank et al., 2017).

Structural Equation Modelling (SEM) was conducted to identify the strength of each path of the hypothetical model (see Fig. 1) and verify the suitability of the conceptual model. Covariance-based SEM (CB-SEM) is the most common approach in SEM, and it follows maximum likelihood (ML) estimation procedure and aims to minimise the difference between the observed and estimated covariance matrix (Astrachan et al., 2014). AMOS is widely used for CB-SEM estimation, and as the questionnaire data with a large sample size that fits the normal distribution, this study chose this method of SEM estimation using IBM SPSS AMOS v25.0. CB-SEM was performed here to explain the relationship between household practices, built environment and appliance characteristics with the observed variable of increase in appliance ownership after shifting in the slum rehabilitation housing. Global fit indices were reported as per the work of Fu et al. (2019) to check the reliability of the model (Model fit results are shown in Table 2 in Section 4.2).

## 4 Result and discussion

### 4.1 Descriptive of appliance ownership and household practice

**Fig. 2:**
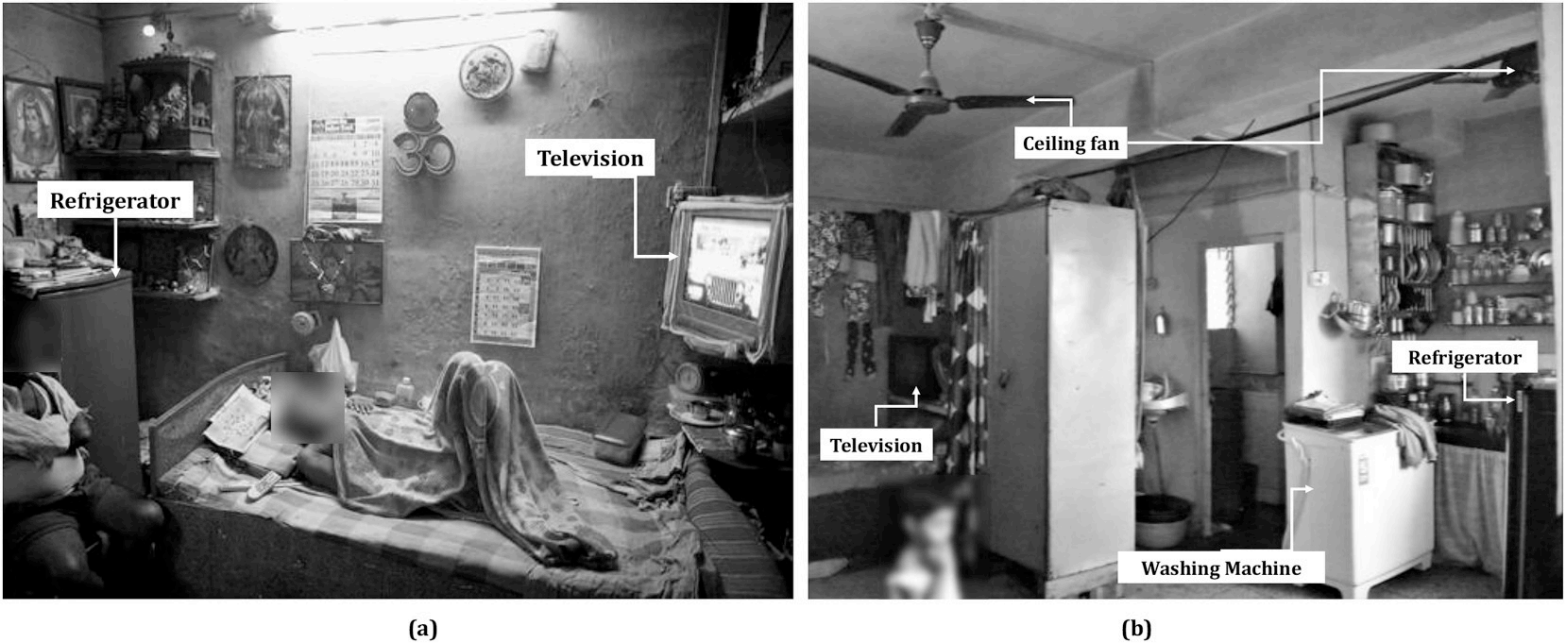
(a) Household appliances in a typical (a) horizontal slum house (80 sq ft); (b) slum rehabilitation house (265 sq ft)Source: (a) Daniel Berehulak/Getty Images Europe (b) Authors'.

The socio-economic conditions of the occupants remained the same despite whether they lived in horizontal slums or the vertical apartment of the slum rehabilitation housing (SRH). It remains a salient feature of this study that the effect of low-income built environment transition can influence household electricity demand through a change of household practices. The typical appliance layout of horizontal slums and SRH apartment units are illustrated in Fig. 2a and b, respectively. In horizontal slums, the occupants had access to electricity and appliances like TVs and refrigerators just like that of the SRH housing. However, smaller floor area in the horizontal slums (∼80 sq ft) restricted higher uptake of the appliance, and their household practices were performed outdoors in a communal way. Common practices like washing clothes, cooking, cleaning and socialising with neighbours were all performed in outdoor community spaces in the slums. As they moved into vertical SRH apartments, they were provided with relatively higher floor area (∼265 sq ft) that may have influenced purchasing decisions of household appliances. It is further influenced by the aspirations of occupants to climb up the social ladder, as permanent housing structure in Mumbai is a social-status indicator (Sunikka-Blank et al., 2019).

**Fig. 3:**
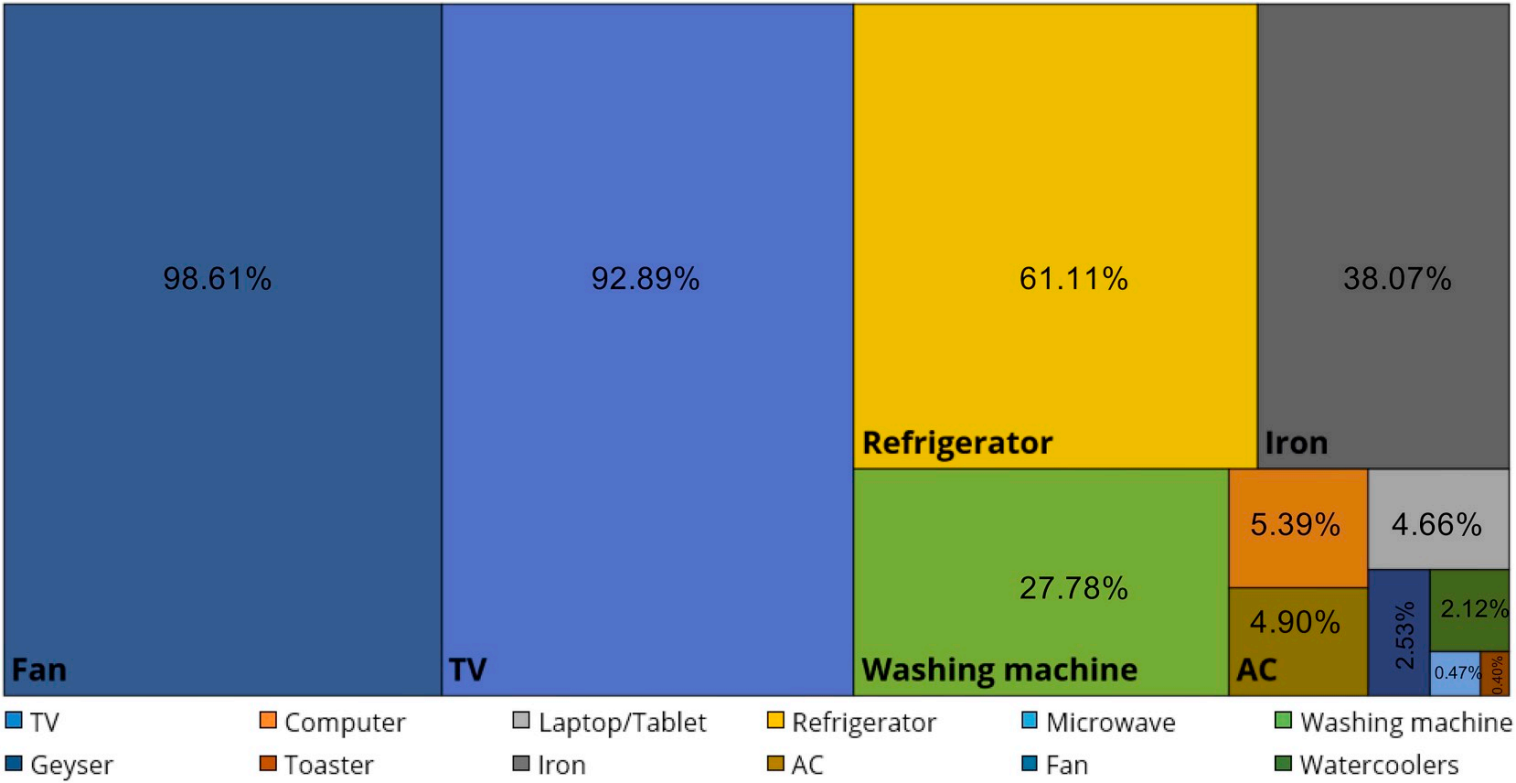
Share of household appliances in the sample size (n= 1224).

**Fig. 4:**
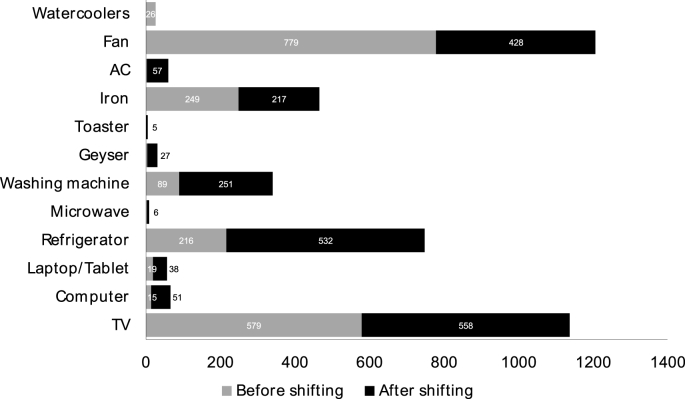
Comparison of household appliance ownership before and after shifting to the slum rehabilitation houses (n = 1224).

The current status of appliance ownership is illustrated in Fig. 3 indicating ceiling fan (98.61%), television (TV) (92.89%) and refrigerator (61.11%) to be a most common household appliance in the study area. Washing machines (27.78%) and cloth irons (38.07%) were present in almost one-third of the surveyed households. In this study, appliances like washing machines, TVs and refrigerators were categorised as energy intensive devices (see Section 3.1). It can be seen from Fig. 4 that most of these energy-intensive devices were purchased after shifting to the SRH. It supports our general hypothesis that the transition of the built environment from horizontal slums to vertical apartments (SRH) influences the purchasing of household appliances that increase the electrical energy intensity of the households.

Based on Fig. 4, the rise in appliance ownership after shifting to SRH is significantly substantial for refrigerators and washing machines. Almost 72% of the refrigerators and 75% of the washing machines were bought after moving into the rehabilitation house (see Fig. 4). Television ownership almost doubled (49%) on moving to SRH, and similar numbers are recorded for an increase in clothing iron ownership (47%). Contrastingly, ownership of air conditioning units (AC) rose by 100% (see Fig. 4), even though AC units represented only 4.90% of total appliance ownership in the study area (See Fig. 3). It is an indicator of growing middle-class and energy-intensive behaviour among the residents.

**Fig. 5:**
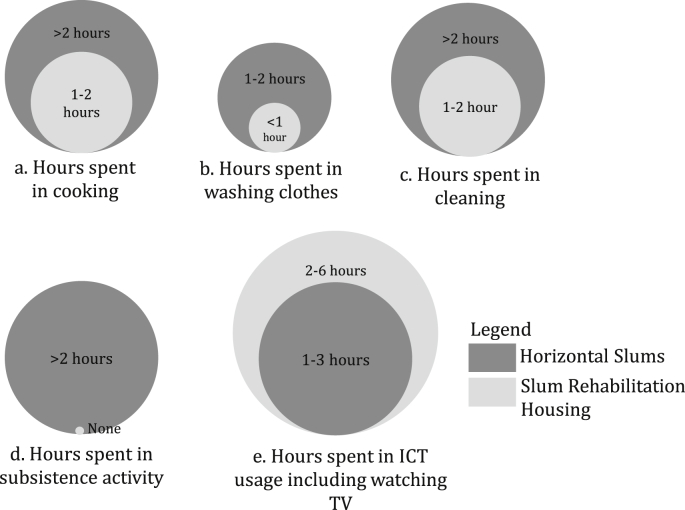
Average time spent in household activities in horizontal slums and slum rehabilitation housing.

While almost 99% of the surveyed households had ceiling fans (see Fig. 3), there was a 35.45% increase in its ownership upon shifting to SRHs. A ceiling fan is the only ‘cooling’ devices available to occupants in the SRHs that is used in the regulation of the occupants' thermal comfort. Increase in its ownership can indicate towards increased discomfort through built environment and is further discussed later in this section concerning the SEM results (see Fig. 5). Increase in ICT device ownerships like computer, TV, laptop/tablet computers can influence household practices. Fig. 4 shows a significant rise in the computer (up by 77%), laptop/tablet (up by 67%) ownership among the occupants along with other devices like toaster (up by 100%), microwave (up by 100%) and geyser (up by 99%) for hot water. These devices account for less than one-tenth the total appliance ownership (see Fig. 3), but their rise in ownership point towards rising energy demand in these low-income areas.

Fig. 5 shows the average time spent in the household activities that constitute the social practices of the occupants in the slums and the SRH. The average time spent in household activities in horizontal slums and SRH has contrasting differences in the organisation of the spaces, as mentioned earlier. The life in the horizontal slum was communal therefore activities like cooking, cleaning and washing clothes used to take a greater share of time (see Fig. 5). On moving to the SRH, life became more private, and household practices reoriented around it. The washing time reduced due to shifting of practices to washing machines, the cooking time went down as they had to cook for only the family members, and not as a communal meal. Indoor and private cooking also influenced higher adoption of refrigerators in the households as they could store a larger quantity of cooked meal for days. It was absent in the horizontal slums because the community preferred readily-cooked meals, which eliminated the requirement of a refrigerator. Lack of space in the horizontal slums was also contributed to the low refrigerator ownership.

The cleaning time also reduced as the occupants had to cater to their own 265 square feet rooms rather than cleaning several slum houses and the collective social spaces as in the horizontal slums. This shifting of household practices to private and indoor life in the SRH due to the architectural design of the built environment encouraged adoption of energy intensive appliances like washing machines and refrigerators. It is discussed in detail in section 4.2.

A stark difference is in the time spent in subsistence activities (see Fig. 5d), wherein horizontal slums it used to be a significant component of their social and household practices. These subsistence activities were informal activities that the occupants used to perform in groups to support their livelihood, and it required a considerable amount of social spaces. In the SRH, lack of social spaces restricted this activity which is a significant cause of discomfort and distress as mentioned in (Sunikka-Blank et al., 2019), (Debnath et al., 2019). Additionally, the effect of indoor and private living is evident from the increase in time spent in watching TV and ICT usage in the SRH in comparison to the horizontal slums (see Fig. 5e). The increased TV and ICT usage have a strong influence on the total increase of indoor energy-intensive activities in the SRH that contributes to an increase in the residential electricity bills. These descriptive evidences support our initial observation that change in household practices contributes to higher appliance ownership, where household practices are changed as the slum dwellers are moved from their informal shacks to formal apartments in the SRH. The next section quantitatively demonstrates this nexus and confirms the significant influence of such non-income drivers in determining the residential energy demand in such low-income settlements.

### 4.2 Model estimation for all data

**Fig. 6:**
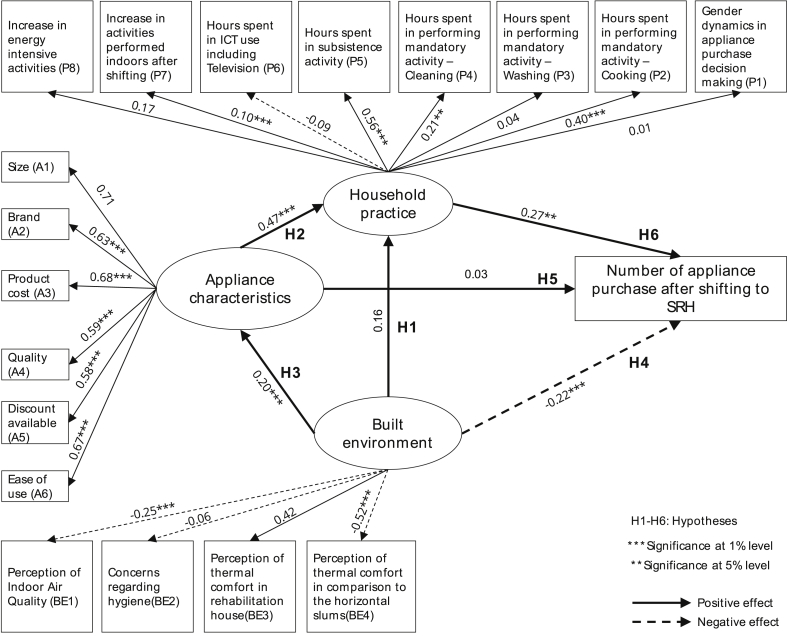
Model estimation using all data (n = 1224). Note: The numbers in the arrows represent factor loadings.

The final SEM model and model fit indices for all data (n = 1224) are shown in Fig. 6 and Table 2, respectively. The model fit well as per the indices and their satisfaction criteria (see Table 2). Overall, the model establishes the latent connections between the effects of low-income built environment under transition from informal structures to permanent vertical apartments (slum rehabilitation housing (SRH)) on changing household practices which ultimately influences higher appliance ownership in slum rehabilitated occupants. The SEM model in Fig. 6, demonstrates quantitatively that non-income factors around energy practices influence appliance ownership, and therefore effect electricity consumption in low-income settlements.

**Table 2:** Model-fit results of the final model.

	Indicators		Criteria	Results
**Absolute fit measures**	χ^2^/df (cmin/df)	Chi-square/degree of freedom	<3.00	2.735
	RMSEA	Root Mean Square Error of Approximation	<.08	.038
	AGFI	Adjusted Goodness of Fit Index	>.80	.956
	GFI	Goodness of Fit Index	>.80	.972
	SRMR	Standard Root Mean-square Residual	<.08	.018
**Incremental fit measures**	NFI	Normed Fit Index	>.90	.932
	CFI	Comparative Fit Index	>.90	.955
	IFI	Incremental fit index	>.90	.956
	TLI	Tucker Lewis Index	>.90	.935
**Parsimonious fit measures**	PGFI	Parsimonious Goodness of Fit Index	>.50	.690
	PNFI	Parsimonious Normed Fit Index	>.50	.643
	PCFI	Parsimonious Comparative Fit Index	>.50	.659

Non-income factors chosen for this study was represented through six hypotheses illustrated in Fig. 1. Hypothesis H1, H2 and H3 constituted the structural model linking the influence of built environment on appliance characteristics and changing household practices (see Fig. 6). Model results show that change of built environment from horizontal slums to vertical SRH apartments positively influenced household practices (0.16), while it significantly accentuated on specific appliance characteristics (0.20, p < 0.01) to be central in purchase decision making (see Fig. 6). The appliance characteristics have a significant positive influence on the changing household practices in the SRH (0.47, p < 0.01).

Specific appliance characteristics that have a significant positive effect on the household practices of the occupants are brand (0.63, p < 0.01), product cost (0.68, p < 0.01), quality (0.59, p < 0.01), discount available (0.58, p < 0.01) and ease of use (0.67, p < 0.01) (see Fig. 6). As mentioned in section 3.2, these variables became important for the occupants on moving to the permanent houses in the SRH because they strongly consider the shift as a step up the social ladder. It further encourages the purchase of rated appliances that have these specific characteristics to suit their aspirational element of ‘owning permanent housing in the city’. In the horizontal slums, the occupants preferred buying unrated appliances to save on the upfront cost (Khosla and Bharadwaj, 2015). While it is a good indicator from the perspectives of energy efficiency policies that consumers are voluntarily moving towards rated appliances, but this segment of consumers have the added burden of poverty as slum rehabilitation did not change their socio-economic conditions. Therefore, these characteristics do not have a significant influence on a high number of appliance ownership (0.03) on shifting from slums to SRH (see Fig. 6). Instead, these appliance characteristics strongly influence household practices that in turn effects appliance purchase decision in the SRH.

The change of the built environment influences changes in household practices (see Fig. 6), and it supports the hypothesis H1. The change in the built environment is characterised by the difference in architectural designs between horizontal slum communities and the SRH apartments. As described in section 3.2, this change is primarily characterised by the lack of outdoor social spaces in the SRH. Household practices in the horizontal slums revolved around these outdoor spaces where mandatory activities like cooking, cleaning and washing clothes used to be performed communally. The current design of the SRH restricts such as social spaces (Sunikka-Blank et al., 2019), which is shifting these activities indoors and is altering the household practices. Increase in indoor activities is increasing the energy intensity of the activities that are translated into higher electricity bills for these low-income households (Debnath et al., 2019). It points toward the critical nexus between housing design and energy choices in such low-income settlement programs that can aid in deriving effective energy policies for people living in poverty.

Model results show that there is a significant increase in indoor household activities upon moving into the SRH that significantly influences the household practices (0.10, p < 0.01) that in turn strongly influences higher appliance ownership (0.27, p < 0.05) (see Fig. 6). Increase in energy-intensive activities on shifting to the SRH has a sizable factor loading on the household practices (0.17). It is due to private usage of appliances in contrast to that of the slums.

The hypothesis H4 relates to the relationship between the built environment and uptake of appliances (see Fig. 6 and section 3.2). The significant negative factor loading (0.22, p < 0.01) can be interpreted as certain aspects of built environment deteriorate upon moving into rehabilitation housing which influences the purchasing of appliances to compensate for this loss in home comfort. Poor built environment condition in the SRH is a factor of its inefficient design that negatively effects the thermal comfort (0.52, p < 0.01) and indoor air quality (0.25, p < 0.01). As the household practices have moved indoors in the SRH, poor thermal comfort and indoor air quality further contribute to occupants’ discomfort and distress (Debnath et al., 2019). It, in turn, leads to uptake of appliances that can relieve them from the added discomfort.

Considering the direct and indirect effects on the appliance purchase after shifting, the total estimated effect of each factor is shown in Table 3. Appliance characteristics (0.158) and household practice (0.265) had a stronger total effect on the number of appliance ownership after shifting to the SRH. The built environment had a stronger indirect effect (0.75) on the increased appliance ownership after shifting.

**Table 3:** Effect of each factor on the number of appliance ownership after shifting.

	Total effect	Direct effect	Indirect effect
Built environment	-.149	-.224	0.75
Appliance characteristics	.158	.033	0.125
Household practice	.265	.265	0.000

### 4.3 Inference of the causal linkages

The empirical results presented in section 4.2 demonstrates the significant linkages between household practice and appliance characteristics with increased appliance ownership in the study area (see Fig. 5). Change of practices like confining daily activities indoors was found to have a significant association with the increase in energy-intensive activities (see Fig. 6) like washing clothes in washing machines, indulging in more ICT usage and increased refrigerator usage and ownership as compared to the horizontal slums (see Fig. 4). Built environment elements like lack of social spaces was a critical confounding factor that mediated higher appliance ownership through a change of practices.

The household practices mainly performed by women in the horizontal slums were an open system where spatially unbounded multitasking of activities occurred. For example, women cooked and washed clothes outdoors while socialising with their neighbours. The traditional practice of preparing fresh meals along with ease of availability of fresh vegetables from the local ‘*bazaar*’ dismissed the need for refrigerators. The ineffective design of SRH with low accessibility made the activities singular and space-bound, triggering energy-intensive indoor living. Presently, refrigerator and TV are the most common appliance that was purchased after shifting to the SRH (see Fig. 4) to compensate for the discomforts from the poor built environment (Debnath et al., 2019). The poor indoor air quality, thermal discomfort and lack of hygiene in the SRH are eased through higher appliance ownership which lead to higher electricity demand. If appliance ownership is assumed to have a linear association with energy demand, then this evidence suggests that energy demand will rise multi-fold with the future building stock. Understanding such built environment induced energy demand becomes more important as 70% of India's low-middle income building stocks is yet to be built (Bardhan and Debnath, 2016).

The results from this study are particularly relevant in designing better energy policies for low-income housing sector through regulatory changes. The policies should be in tandem with the quality of the built environment and appliance purchase behaviour of appliances like refrigerators and washing machines that contribute sufficiently to household welfare by reducing female burden in these low-income habitats. Dhanaraj et al. (2018), regarded these appliances as ‘welfare appliances’. Thus, a ‘good’ energy policy for such low-income houses should enable the usage of these welfare appliances through built environment design regulations or by providing economic incentives for owning them.

## 5 Conclusion and Policy implications

We have examined the drivers of higher appliance ownership in low-income settlements that is undergoing transition. This study demonstrates quantitatively that non-income factors around energy practices influence appliance ownership, and therefore electricity consumption. We find that appliance ownership increases when household practices shift indoors. We also find that poor indoor air quality, hygiene and thermal comfort act as a compensatory trigger for higher appliance purchase. Sub-standard design of social housing like the slum rehabilitation housing, not only pose health hazards to the occupants, but our empirical findings indicate the possibility of energy burden on the occupants through higher electricity bills. The findings of this study are crucial for the quantification of a practice-based approach to make ‘good’ energy policies. Good energy policy in this context should provide the drive for better-built environment design and higher inclusion of welfare appliance to deliver energy efficiency naturally in changing household practices. For example, better-designed SRH can be pre-fitted with energy labelled welfare appliances that can naturally embed relative energy savings in changing practices. This study opens a new dialogue on the inclusion of the effects of changing practices in search of sound energy policy for the Global South.

The significant implications of this study lie in contributing to the energy demand forecasting for growing megacity like Mumbai. It is primarily because the occupants of low-income housing are assumed to belong to the lowest strata of the energy ladder, i.e. they will consume the least energy. However, this study has shown that non-income factors like a change in household practices and built environment characteristics can significantly increase the energy demand despite low-income status. This finding emphasises on the need of designing policies by considering the effects of housing and energy choices of people living in low and middle-income social classes. It remains a significant gap in the current policy discussions as the low-income population are assumed to consume the least energy and resources are allocated accordingly. Such gap if not addressed can pose threats to India's energy security, especially when two-thirds of the building stocks are yet to be built. While India will pull-out millions of its citizens from extreme poverty in the coming decades, the future of urbanisation will primarily belong to the low-income strata. Hence, understanding their practices and energy choices will be critical in determining future energy sustainability.

## Funding

RD is supported by Bill & Melinda Gates Foundation (Grant no. OPP1144) through the Gates-Cambridge Scholarship. This study is in parts funded by Ministry of Human Resource Development, Government of India (Grant no. 14MHRD005) under the Frontier Areas in Science and Technology grant awarded to RB.

## Footnotes

1 Mandatory activities are those household activities which are compulsory for daily living. This nomenclature is borrowed from activity-based approach studies (Chikaraishi et al., 2017).

## References

[bib1] Astrachan C.B., Patel V.K., Wanzenried G. (2014). A comparative study of CB-SEM and PLS-SEM for theory development in family firm research. J. Fam. Bus. Strateg..

[bib2] Auffhammer M., Wolfram C.D. (2014). Powering up China: income distributions and residential electricity consumption. Am. Econ. Rev..

[bib3] Bardhan R., Debnath R. (2016). Towards daylight inclusive bye-law: daylight as an energy saving route for affordable housing in India. Energy Sustain. Dev..

[bib4] Bardhan R., Sarkar S., Jana A., Velaga N.R. (2015). Mumbai slums since independence: evaluating the policy outcomes. Habitat Int..

[bib5] Bardhan R., Debnath R., Malik J., Sarkar A. (2018). Low-income housing layouts under socio-architectural complexities: a parametric study for sustainable slum rehabilitation. Sustain. Cities Soc..

[bib6] Bardhan R., Debnath R., Jana A., Norford L.K. (2018). Investigating the association of healthcare-seeking behavior with the freshness of indoor spaces in low-income tenement housing in Mumbai. Habitat Int..

[bib7] Batih H., Sorapipatana C. (2016). Characteristics of urban households' electrical energy consumption in Indonesia and its saving potentials. Renew. Sustain. Energy Rev..

[bib8] Bhattacharyya S.C. (2015). “Influence of India's transformation on residential energy demand. Appl. Energy.

[bib9] Bisaga I., Parikh P. (2018). To climb or not to climb? Investigating energy use behaviour among Solar Home System adopters through energy ladder and social practice lens. Energy Res. Soc. Sci..

[bib10] Brounen D., Kok N., Quigley J.M. (2012). Residential energy use and conservation: economics and demographics. Eur. Econ. Rev..

[bib11] Cabeza L.F., Urge-Vorsatz D., McNeil M.A., Barreneche C., Serrano S. (2014). Investigating greenhouse challenge from growing trends of electricity consumption through home appliances in buildings. Renew. Sustain. Energy Rev..

[bib12] Chikaraishi M., Jana A., Bardhan R., Varghese V., Fujiwara A. (2017). A framework to analyze capability and travel in formal and informal urban settings: a case from Mumbai. J. Transp. Geogr..

[bib13] Daioglou V., van Ruijven B.J., van Vuuren D.P. (2012). Model projections for household energy use in developing countries. Energy.

[bib14] Debnath R. (2019). Invisible drivers of energy use in poverty. Gates Day of Research.

[bib15] Debnath R. (2019). How does slum rehabilitation influence energy use in poverty?. Churchill College Conference on Everything.

[bib16] Debnath R., Bardhan R., Jain R.K. (2017). A data-driven and simulation approach for understanding thermal performance of slum redevelopment in Mumbai, India. Proceeding of the 15th IBPSA Conference.

[bib17] Debnath R., Bardhan R., Sunikka-Blank M. (May 2019). Discomfort and distress in slum rehabilitation: investigating a rebound phenomenon using a backcasting approach. Habitat Int..

[bib18] Dhanaraj S., Mahambare V., Munjal P. (2018). From income to household welfare: lessons from refrigerator ownership in India. J. Quant. Econ..

[bib19] Eckhardt G.M., Mahi H. (2012). Globalization, consumer tensions, and the shaping of consumer culture in India. J. Macromarketing.

[bib20] Ekholm T., Krey V., Pachauri S., Riahi K. (2010). Determinants of household energy consumption in India. Energy Policy.

[bib21] Farsi M., Filippini M., Pachauri S. (2007). Fuel choices in urban Indian households. Environ. Dev. Econ..

[bib22] Filippini M., Pachauri S. (2004). Elasticities of electricity demand in urban Indian households. Energy Policy.

[bib23] Foulds C., Powell J., Seyfang G. (2016). How moving home influences appliance ownership: a Passivhaus case study. Energy Effic..

[bib24] Fu B., Kurisu K., Hanaki K., Che Y. (2019). Influential factors of public intention to improve the air quality in China. J. Clean. Prod..

[bib25] Halvorsen B., Larsen B.M. (2001). Norwegian residential electricity demand-a microeconomic assessment of the growth from 1976 to 1993. Energy Policy.

[bib26] IEA (2009). “Gadgets and Gigawatts. Policies for Energy Efficient Electronics,” Paris, France.

[bib27] Janda K.B. (2011). “Buildings don't use energy: people do. Architect. Sci. Rev..

[bib28] Jones R.V., Fuertes A., Lomas K.J. (2015). The socio-economic, dwelling and appliance related factors affecting electricity consumption in domestic buildings. Renew. Sustain. Energy Rev..

[bib29] Kemmler A. (2007). Factors influencing household access to electricity in India. Energy Sustain. Dev..

[bib30] Khalid R., Sunikka-Blank M. (2017). Homely social practices, uncanny electricity demands: class, culture and material dynamics in Pakistan. Energy Res. Soc. Sci..

[bib31] Khalid R., Sunikka-Blank M. (2018). Evolving houses, demanding practices: a case of rising electricity consumption of the middle class in Pakistan. Build. Environ..

[bib32] Khosla R., Bharadwaj A. (2015). Plugging in: Electricity Consumption in Indian Homes. http://www.prayaspune.org/peg/appliances-used-in-affordable-housing.

[bib33] Kowsari R., Zerriffi H. (2011). Three dimensional energy profile:. A conceptual framework for assessing household energy use. Energy Policy.

[bib34] Labanca N., Bertoldi P. (2018). Beyond energy efficiency and individual behaviours: policy insights from social practice theories. Energy Policy.

[bib35] Larsen B.M., Nesbakken R. (2004). Household electricity end-use consumption: results from econometric and engineering models. Energy Econ..

[bib36] Leahy E., Lyons Sean S. (2010). Energy use and appliance ownership in Ireland. Energy Policy.

[bib37] Louw K., Conradie B., Howells M., Dekenah M. (2008). Determinants of electricity demand for newly electrified low-income African households. Energy Policy.

[bib38] McNeil M.A., Letschert V.E. (2010). Modeling diffusion of electrical appliances in the residential sector. Energy Build..

[bib39] McNeil M.A., Letschert V.E., de la Rue du S., Can, Ke J. (2013). Bottom-Up Energy Analysis System (BUENAS)-an international appliance efficiency policy tool. Energy Effic..

[bib40] Nair A. (21-Sep-2018). SRA Houses 40 Per Cent Illegal Tenants.

[bib41] Nijman J. (2015). “India's urban future: views from the slum. Am. Behav. Sci..

[bib42] Nutkiewicz A., Jain R.K., Bardhan R. (2018). Energy modeling of urban informal settlement redevelopment: exploring design parameters for optimal thermal comfort in Dharavi, Mumbai, India. Appl. Energy.

[bib43] O'Doherty J., Lyons S., Tol R.S.J. (2008). Energy-using appliances and energy-saving features: determinants of ownership in Ireland. Appl. Energy.

[bib44] Pothitou M., Hanna R.F., Chalvatzis K.J. (2017). “ICT entertainment appliances' impact on domestic electricity consumption. Renew. Sustain. Energy Rev..

[bib45] Rao N.D., Ummel K. (2017). White goods for white people? Drivers of electric appliance growth in emerging economies. Energy Res. Soc. Sci..

[bib46] Reckwitz A. (2002). Toward a theory of social practices; a development in culturalist theorizing. Eur. J. Soc. Theory.

[bib47] Rong Z., Yao Y. (2003). Public service provision and the demand for electric appliances in rural China. China Econ. Rev..

[bib48] Røpke I. (2009). Theories of practice - new inspiration for ecological economic studies on consumption. Ecol. Econ..

[bib49] Røpke I., Christensen T.H. (2012). Energy impacts of ICT - insights from an everyday life perspective. Telematics Inf..

[bib50] Shove E., Pantzar M., Watson M. (2012). The Dynamics of Social Practice : Everyday Life and How it Changes.

[bib51] Sunikka-Blank M., Galvin R., Behar C. (2017). Harnessing social class, taste and gender for more effective policies. Build. Res. Inf..

[bib52] Sunikka-Blank M., Bardhan R., Haque A. (2019). Gender, domestic energy and design of inclusive low-income habitats: a case of slum rehabilitation housing in Mumbai. Energy Res. Soc. Sci..

[bib53] Tiwari P. (2000). Architectural, demographic, and economic causes of electricity consumption in Bombay. J. Policy Model..

[bib54] Van Der Kroon B., Brouwer R., Van Beukering P.J.H. (2013). The energy ladder: theoretical myth or empirical truth? Results from a meta-analysis. Renew. Sustain. Energy Rev..

[bib55] Wolfram C., Shelef O., Gertler P. (2012). How will energy demand develop in the developing world?. J. Econ. Perspect..

[bib56] Wyatt P. (2013). A dwelling-level investigation into the physical and socio-economic drivers of domestic energy consumption in England. Energy Policy.

[bib57] Zhang Y. (2016). Building a slum-free Mumbai. Case Study.

